# Intermittent versus continuous oxaliplatin and fluoropyrimidine combination chemotherapy for first-line treatment of advanced colorectal cancer: results of the randomised phase 3 MRC COIN trial

**DOI:** 10.1016/S1470-2045(11)70102-4

**Published:** 2011-06-04

**Authors:** Richard A Adams, Angela M Meade, Matthew T Seymour, Richard H Wilson, Ayman Madi, David Fisher, Sarah L Kenny, Edward Kay, Elizabeth Hodgkinson, Malcolm Pope, Penny Rogers, Harpreet Wasan, Stephen Falk, Simon Gollins, Tamas Hickish, Eric M Bessell, David Propper, M John Kennedy, Richard Kaplan, Timothy S Maughan, on behalf of the MRC COIN Trial Investigators

**Affiliations:** a School of Medicine, Cardiff University, Cardiff, UK; b Medical Research Council Clinical Trials Unit, London, UK; c St James's University Hospital and University of Leeds, Leeds, UK; d Queens University Belfast, Belfast, UK; e Weston Park Hospital, Sheffield, UK; f Patient Representative, Velindre Cancer Centre, Cardiff, UK; g Charing Cross Hospital, London, UK; h Hammersmith Hospital, London, UK; i Bristol Haematology and Oncology Centre, Bristol, UK; j Glan Clwyd Hospital, Rhyl, UK; k Bournemouth and Poole Hospitals and Bournemouth University, Bournemouth, UK; l Nottingham City Hospital, Nottingham, UK; m St Bartholomew's Hospital, London, UK; n ICORG, All Ireland Co-operative Oncology Research Group, Dublin, Ireland

## Abstract

**Background:**

When cure is impossible, cancer treatment should focus on both length and quality of life. Maximisation of time without toxic effects could be one effective strategy to achieve both of these goals. The COIN trial assessed preplanned treatment holidays in advanced colorectal cancer to achieve this aim.

**Methods:**

COIN was a randomised controlled trial in patients with previously untreated advanced colorectal cancer. Patients received either continuous oxaliplatin and fluoropyrimidine combination (arm A), continuous chemotherapy plus cetuximab (arm B), or intermittent (arm C) chemotherapy. In arms A and B, treatment continued until development of progressive disease, cumulative toxic effects, or the patient chose to stop. In arm C, patients who had not progressed at their 12-week scan started a chemotherapy-free interval until evidence of disease progression, when the same treatment was restarted. Randomisation was done centrally (via telephone) by the MRC Clinical Trials Unit using minimisation. Treatment allocation was not masked. The comparison of arms A and B is described in a companion paper. Here, we compare arms A and C, with the primary objective of establishing whether overall survival on intermittent therapy was non-inferior to that on continuous therapy, with a predefined non-inferiority boundary of 1·162. Intention-to-treat (ITT) and per-protocol analyses were done. This trial is registered, ISRCTN27286448.

**Findings:**

1630 patients were randomly assigned to treatment groups (815 to continuous and 815 to intermittent therapy). Median survival in the ITT population (n=815 in both groups) was 15·8 months (IQR 9·4–26·1) in arm A and 14·4 months (8·0–24·7) in arm C (hazard ratio [HR] 1·084, 80% CI 1·008–1·165). In the per-protocol population (arm A, n=467; arm C, n=511), median survival was 19·6 months (13·0–28·1) in arm A and 18·0 months (12·1–29·3) in arm C (HR 1·087, 0·986–1·198). The upper limits of CIs for HRs in both analyses were greater than the predefined non-inferiority boundary. Preplanned subgroup analyses in the per-protocol population showed that a raised baseline platelet count, defined as 400 000 per μL or higher (271 [28%] of 978 patients), was associated with poor survival with intermittent chemotherapy: the HR for comparison of arm C and arm A in patients with a normal platelet count was 0·96 (95% CI 0·80–1·15, p=0·66), versus 1·54 (1·17–2·03, p=0·0018) in patients with a raised platelet count (p=0·0027 for interaction). In the per-protocol population, more patients on continuous than on intermittent treatment had grade 3 or worse haematological toxic effects (72 [15%] *vs* 60 [12%]), whereas nausea and vomiting were more common on intermittent treatment (11 [2%] *vs* 43 [8%]). Grade 3 or worse peripheral neuropathy (126 [27%] *vs* 25 [5%]) and hand–foot syndrome (21 [4%] *vs* 15 [3%]) were more frequent on continuous than on intermittent treatment.

**Interpretation:**

Although this trial did not show non-inferiority of intermittent compared with continuous chemotherapy for advanced colorectal cancer in terms of overall survival, chemotherapy-free intervals remain a treatment option for some patients with advanced colorectal cancer, offering reduced time on chemotherapy, reduced cumulative toxic effects, and improved quality of life. Subgroup analyses suggest that patients with normal baseline platelet counts could gain the benefits of intermittent chemotherapy without detriment in survival, whereas those with raised baseline platelet counts have impaired survival and quality of life with intermittent chemotherapy and should not receive a treatment break.

**Funding:**

Cancer Research UK.

## Introduction

The treatment of advanced colorectal cancer has improved substantially during the past decade with the introduction of new and more effective drugs and advances in our understanding of the disease's molecular biology. For many patients, these developments have changed the therapeutic perspective of advanced colorectal cancer from an acute to a chronic condition. Despite increasing use of surgery for metastatic disease, treatment remains palliative in intent for most patients. Therefore, from diagnosis onwards, an individual might spend most of their remaining life continuously on therapy, with the associated toxic effects, clinic attendances, detriment to quality of life, and expense. Strategies that minimise time on treatment, reduce side-effects, and improve quality of life, while maintaining duration of survival, are highly desirable.

Before the introduction of irinotecan and oxaliplatin into treatment schedules for advanced colorectal cancer, fluorouracil-based chemotherapy was generally continued until unacceptable toxic effects or disease progression. This approach has continued, with few trials examining alternative strategies. Most ongoing study protocols for first-line treatment of advanced colorectal cancer require a minimum of 6 months of continuous therapy for both standard and experimental arms. The resultant cumulative sensory neuropathy with oxaliplatin and hand–foot syndrome with fluoropyrimidines^1^ adversely affect quality of life and overall dose intensity because of reduced dosing in subsequent treatment cycles.

One potential method to reduce toxic effects and improve quality of life is to consider alternatives to continuous chemotherapy. Intermittent therapy given for a restricted period and then restarted, either after a predefined interval (predefined treatment) or at disease progression (repeat therapy), are such alternatives. Concerns remain that intermittent therapy in cancer could reduce tumour control or promote resistance to treatment. However, in breast and prostate cancer, intermittent hormones or cytotoxic agents have not reduced median overall survival.^2^, ^3^, ^4^

A previous Medical Research Council (MRC) trial, CR06B, assessed 354 patients with advanced colorectal cancer treated with the de Gramont (fluorouracil and folinic acid) schedule, continuous infusional fluorouracil, or raltitrexed.^5^ Those with stable or responding disease at 12 weeks were further randomly assigned to continue therapy until progressive disease or to stop, with the option to restart the same chemotherapy at later progression. There was no evidence of a difference in overall survival between the two strategies (hazard ratio [HR] 0·87, 95% CI 0·69–1·09, p=0·23), with the results even slightly favouring intermittent over continuous treatment. Patients on intermittent chemotherapy had significantly fewer toxic effects and serious adverse events than did those on continuous chemotherapy. Although criticised for its small size and failure of randomly assigned patients to restart protocol treatment, this trial gave impetus to the notion of intermittent therapy in advanced colorectal cancer and set a precedent for further trials of intermittent combination chemotherapy.^6^

With its cumulative sensory neuropathy, oxaliplatin is an appropriate drug to use in the exploration of intermittent therapy and has been assessed in two trials, OPTIMOX-1^7^ and OPTIMOX-2.^8^ In OPTIMOX-1,^7^ continuous oxaliplatin and fluorouracil were compared with a novel strategy of planned oxaliplatin breaks, but with continuous fluorouracil. In OPTIMOX-2,^8^ the OPTIMOX-1 intermittent oxaliplatin strategy was compared with a complete chemotherapy-free interval strategy. Neither trial showed a significant reduction in survival with intermittent therapy, although OPTIMOX-2 showed a trend in favour of continuation of fluorouracil during oxaliplatin breaks, and has been adopted as the benchmark for standard practice. This outcome is despite failure to recruit beyond phase 2 after the licensing of bevacizumab and that the trend in favour of continuous treatment was not statistically significant.

The phase 3 COIN trial (COntinuous or INtermittent) was developed to conclusively address this issue. We report here the results of this trial, to our knowledge the largest trial of intermittent versus continuous combination chemotherapy in advanced colorectal cancer. The COIN trial also assessed the effect of addition of cetuximab to continuous oxaliplatin and fluoropyrimidine combination chemotherapy; the results of this comparison are reported in a companion paper.^9^

## Methods

### Study design and participants

The full protocol can be found on the MRC Clinical Trials Unit website. Patients were recruited by consultant oncologists at sites in the UK and Ireland that routinely undertake treatment of advanced colorectal cancer. Eligibility included written informed consent, age of at least 18 years, and histologically confirmed adenocarcinoma of the colorectum, inoperable metastatic or locoregional measurable disease (Response Evaluation Criteria In Solid Tumors [RECIST] version 1.0),^10^ no previous chemotherapy for metastatic disease, WHO performance status 0–2, and good end-organ function. Patients were excluded if they had previous or present malignant disease, uncontrolled medical comorbidity likely to interfere with COIN treatment or response assessment, known brain metastases, or previous oxaliplatin exposure.

COIN was approved by the UK Medicines and Healthcare Regulatory Agency in June, 2004, and southwest multicentre research ethics committee in December, 2004. Approvals for the Irish sites were obtained from the Irish Medicines Board in November, 2006, and St James's Hospital/Adelaide & Meath Hospital, Incorporating the National Children's Hospital research ethics committee in December, 2006. The trial was coordinated by the MRC Clinical Trials Unit following the principles of International Conference on Harmonisation Good Clinical Practice guidelines, undertaken with a trial management group, monitored at regular intervals by an independent data monitoring committee, and overseen by an independent trial steering committee.

### Randomisation and masking

**Figure 1:**
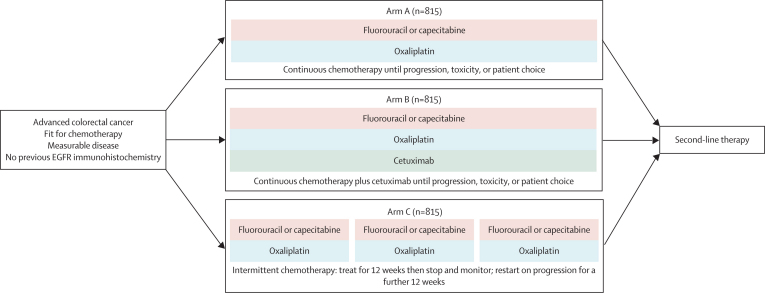
Trial design

Central telephone randomisation was done by the MRC Clinical Trials Unit, using the method of minimisation with a random element. The minimisation factors were hospital, WHO performance status, chemotherapy regimen, previous adjuvant chemotherapy, liver metastases, and peritoneal metastases. Patients were randomly assigned (1:1:1) to the control arm of continuous oxaliplatin-based (oxaliplatin plus capecitabine or oxaliplatin plus fluorouracil and folinic acid) chemotherapy (arm A) or one of two research arms: continuous chemotherapy plus cetuximab (arm B) or intermittent chemotherapy (arm C; figure 1). Treatment allocation was not masked.

### Procedures

Oncologists chose between the two chemotherapy regimens according to local hospital policy or patient preference. Oxaliplatin plus capecitabine was given as a 3-weekly regimen of intravenous oxaliplatin 130 mg/m^2^ over 2 h followed by oral capecitabine 1000 mg/m^2^ twice a day for 2 weeks. Oxaliplatin plus fluorouracil and folinic acid was given as a 2-weekly regimen of intravenous L-folinic acid 175 mg or D,L-folinic acid 350 mg over 2 h given concurrently with oxaliplatin 85 mg/m^2^ over 2 h, followed by intravenous bolus fluorouracil 400 mg/m^2^, and finally fluorouracil 2400 mg/m^2^ infusion over 46 h via an ambulatory pump.

In arm A, treatment was continued until RECIST-defined progressive disease, development of cumulative toxic effects (precluding both oxaliplatin and fluoropyrimidine use), or patients chose to stop. Patients on arm C received chemotherapy for 12 weeks, after which treatment was stopped completely and the patients were assessed. Patients with progressive disease came off protocol treatment and those with stable or responding disease began a complete chemotherapy-free interval. Because the primary endpoint was overall survival, responses were not confirmed by repeat scans and external radiological review was not undertaken. During their chemotherapy-free interval, patients were assessed clinically at least every 6 weeks and radiologically every 12 weeks (or sooner if clinically indicated). The intermittent strategy was that the same chemotherapy was to be restarted on confirmation of progressive disease (with this scan becoming the new baseline measurement). Repeated chemotherapy cycles and chemotherapy-free intervals were continued until progressive disease on treatment was documented or patients chose to stop protocol therapy. Detailed dose reduction and delay protocols were provided (see protocol).

Patient symptoms were assessed by investigators throughout treatment and scored with National Cancer Institute Common Toxicity Criteria for Adverse Events (version 3.0).^11^ Serious adverse events and deaths, together with an assessment of causality, were continuously reported and these were further assessed by an expert practising oncologist on behalf of the MRC.

Patients completed the European Organisation for Research and Treatment of Cancer quality-of-life core questionnaire (QLQ-C30)^12^ and five additional questions (with similar structure to that used in the QLQ-C30) about trial-specific symptoms and patient views on how acceptable and worthwhile their treatment was. Forms were scheduled to be completed at baseline (before randomisation), 6 weeks, 12 weeks, and every 12 weeks thereafter (before knowledge of CT scan results).

The primary objective of this part of the COIN trial was to establish whether intermittent therapy was non-inferior to continuous therapy in terms of overall survival in patients with advanced colorectal cancer. The secondary aims were to evaluate failure-free survival (of the treatment strategies), response, toxic effects, quality of life, and cost-effectiveness. Results of the cost-effectiveness analysis are not yet available. The primary quality-of-life outcome measures were palliation, toxic effects, functional scales, and global quality of life.

### Statistical analysis

The sample size for comparison of arm A versus C was 1614 patients (807 per arm). In the UK-based FOCUS trial,^1^ patients receiving continuous chemotherapy had 2-year overall survival of 20% and a median overall survival of 15·4 months. To show non-inferiority with a one-sided log-rank test with α of 0·1 and power of 90%, 1420 patients would be needed (710 patients per arm). This size would reliably exclude a difference worse than 4·6% in 2-year survival (or >2·15 months in median overall survival). The predefined non-inferiority boundary was 1·162. To account for the roughly 12% of patients who progress or who do not complete 12 weeks of treatment and therefore would not contribute to the comparison, the target sample size was increased to 807 patients per arm, and the analysis was timed to be done when at least 1168 overall survival events had occurred. The primary analysis was one-sided at 90% significance, with the confidence limit calculated as the upper limit of a two-sided 80% CI. All other analyses were two-sided at a 95% significance level. Stata (version 11.1) was used for all statistical analyses.

The primary question was one of non-inferiority, so both intention-to-treat (ITT) and per-protocol analyses of overall survival were planned. The ITT analysis included all patients randomly allocated to treatment groups, whereas the per-protocol analysis included patients who reached the point at which continuous and intermittent strategies diverged—ie, they received a full 12 weeks' treatment (allowing for one missed cycle), remained on trial, and had no evidence of progressive disease at 12 weeks (within 4 weeks). Additionally, the patients included in the per-protocol analysis had to have adhered to the assigned protocol strategy at 12 weeks—ie, patients in arm A continuing treatment and those in arm C starting a chemotherapy-free interval. This population was defined to correspond to the patient cohort who, when seen at the 12-week timepoint (outside a trial context), would benefit from discussion about whether to continue treatment or begin a chemotherapy-free interval.

Overall survival was calculated as time from randomisation to death from any cause. At the time of analysis, survivors were censored at the date that they were last known to be alive. Strategy-failure-free survival was defined as the time from randomisation to failure of the COIN strategy to which a patient was randomly assigned (webappendix p 3). In the continuous treatment group, this measure equated to conventional progression-free survival. In the intermittent treatment group, a strategy-failure-free survival event occurred when a patient had progressive disease during a planned treatment period or within 8 weeks of starting a chemotherapy-free interval. Patients were censored at the last date for which a case report form was completed. The primary analysis timepoint for quality of life was 24 weeks after randomisation, but baseline and 12-week data were also analysed since these timepoints were milestones in the treatment strategy. Only patients in the per-protocol population with data at all three timepoints were analysed. Ordinal logistic regression was used to predict 12-week and 24-week scores rounded into five categories, adjusting for treatment arm and previous scores.

We compared rates of toxic effects between treatment groups using a χ^2^ test, or Fisher's exact test in case of low event rates (n<5). Prognostic and predictive modelling was done with Cox proportional hazards regression. To investigate the effect of prespecified covariates on survival, we built prognostic models using a backward stepwise approach with critical value p=0·05 for both removal from and re-entry into the model at each iteration. To investigate whether these covariates modified the treatment effect, predictive models were fitted that included the covariate, treatment arm, and a treatment-predictive factor interaction term; interaction tests were done with likelihood-ratio tests of the null hypothesis that the interaction coefficient is zero.

This trial is registered, ISRCTN27286448.

### Role of the funding source

The trial was conceived and developed by the National Cancer Research Institute advanced colorectal clinical studies group. The MRC was the overall sponsor of the study, with some responsibilities for the sites in Ireland delegated to the Irish Clinical Oncology Research Group. The MRC Clinical Trials Unit was responsible for all data collection and analysis, and contributed to the writing of the report. The corresponding author had full access to the data and had final responsibility for the decision to submit for publication.

## Results

**Figure 2:**
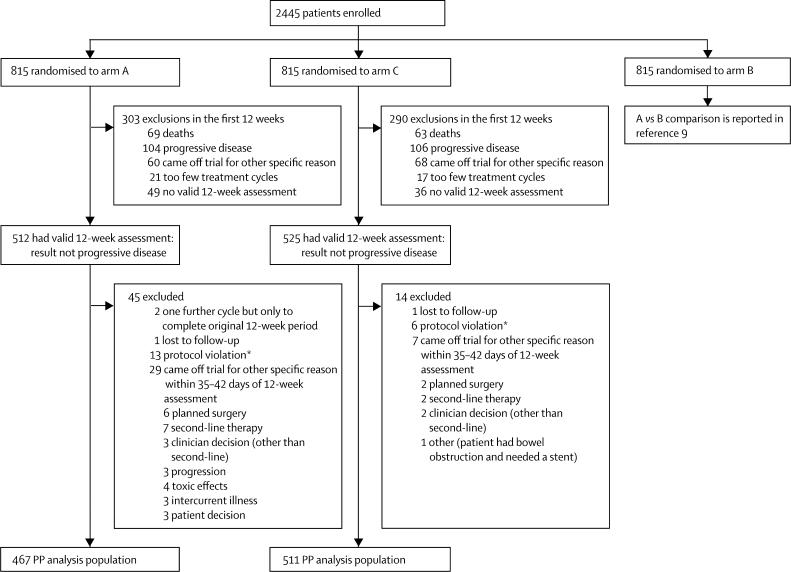
Trial profile PP=per-protocol. *Protocol violation refers to the specific violation of either continuing therapy if in arm C or of stopping therapy if in arm A.

Between March 9, 2005, and May 9, 2008, 2445 patients were randomly allocated to treatment groups at 111 centres in the UK and Ireland, with 1630 patients assigned to the comparison of continuous versus intermittent treatment. Table 1 shows their baseline characteristics, which were well balanced between the trial arms. Figure 2 shows the progress of patients through the stages of the trial. At the time of analysis (database locked Sept 2, 2009), the median duration of follow-up among surviving patients assigned to arm A was 20·9 months (IQR 16·1–28·3) and to arm C was 21·8 months (16·2–29·5). 28 (7%) of 383 surviving patients had no data returned for 12 months and were deemed lost to follow-up.

**Table 1:** Baseline characteristics

	**Arm A (N=815)**	**Arm C (N=815)**
**Choice of chemotherapy at baseline**		
Capecitabine-based	536 (66%)	533 (65%)
Fluorouracil-based	279 (34%)	282 (35%)
**Sex**		
Male	525 (64%)	523 (64%)
Female	290 (36%)	292 (36%)
**Age**		
Median (years)	63 (56–69)	63 (58–70)
≥75 years	74 (9%)	69 (8%)
**WHO performance status**		
0	375 (46%)	375 (46%)
1	378 (46%)	378 (46%)
2	62 (8%)	62 (8%)
**Previous adjuvant chemotherapy**		
None	608 (75%)	608 (75%)
1–6 months	36 (4%)	36 (4%)
>6 months	128 (16%)	131 (16%)
Yes (unspecified)	43 (5%)	40 (5%)
**Site of primary tumour**		
Rectum	243 (30%)	252 (31%)
**Status of primary tumour**		
Resected	445 (55%)	419 (51%)
Unresected	331 (41%)	350 (43%)
Local recurrence	39 (5%)	46 (6%)
**Metastases**		
Metachronous	249 (31%)	241 (30%)
Synchronous	552 (68%)	567 (70%)
Liver only	174 (21%)	179 (22%)
Liver and others	436 (53%)	430 (53%)
Non-liver	198 (24%)	200 (25%)
**Number of metastatic sites**		
One	283 (35%)	284 (35%)
Two	326 (40%)	329 (40%)
More than two	199 (24%)	196 (24%)

Data are n (%) or median (IQR).

35 (2%) patients did not start trial therapy because of clinical deterioration after randomisation, patient choice, or subsequent ineligibility after randomisation. Two-thirds of all patients randomly assigned to treatment groups (536 in arm A, 533 in arm C) received capecitabine-based chemotherapy, and 34% (279 in arm A, 282 in arm C) received fluorouracil-based therapy. No major differences were identified by choice of chemotherapy for any of the outcome measures of this comparison. In the per-protocol population, the geometric mean overall time on treatment in arm C was 5·2 months (95% CI 5·0–5·4) versus 7·5 months (7·3–7·8) in arm A (p<0·0001). Although total dose delivered of chemotherapeutic agents was greater in arm A than in arm C (p<0·0001), dose intensity was greater in arm C during on-treatment periods (p<0·0001). In the intermittent treatment group, 325 (64%) of 511 potentially eligible patients restarted a second 12-week course of chemotherapy after a chemotherapy-free interval.

**Figure 3:**
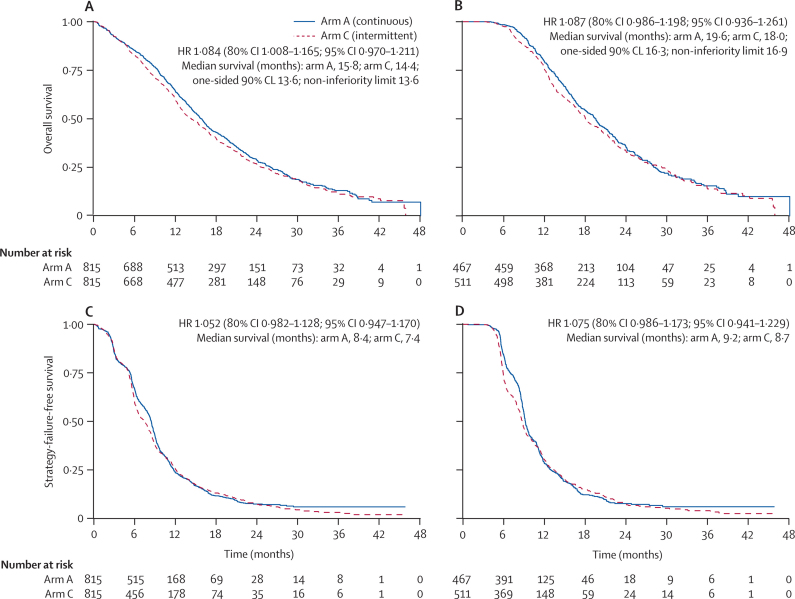
Kaplan-Meier curves for overall survival in (A) the ITT population and (B) the per-protocol population, and strategy-failure-free survival in (C) the ITT population and (D) the per-protocol population Median survival in each arm is derived directly from the Kaplan-Meier curve. Additionally, for overall survival we present median survival in arm C corresponding to the one-sided 90% (ie, upper 80%) confidence limit (CL) of the hazard ratio (HR); and for comparison, the limit of median survival regarded non-inferior with the predefined non-inferiority bound of HR 1·162. This is intended to give a clinical interpretation of the results as compared with the prespecified bound. ITT=intention-to-treat.

1247 (77%) deaths had occurred in the ITT population at the time of analysis. Median survival in the ITT population was 15·8 months (IQR 9·4–26·1) in arm A and 14·4 months (8·0–24·7) in arm C, and in the per-protocol population was 19·6 months (13·0–28·1) in arm A and 18·0 months (12·1–29·3) in arm C. The HR point estimates were 1·084 (80% CI 1·008–1·165) in the ITT population and 1·087 (0·986–1·198) in the per-protocol population; the upper limits were higher than the predefined non-inferiority boundary (figure 3). Kaplan-Meier overall survival in the ITT population for arm A versus arm C was 28·7% versus 26·5% at 2 years and 13·0% versus 11·2% at 3 years.

1166 (94%) of 1247 deaths in the ITT population were due to colorectal cancer. 22 (2%) deaths were reported to be treatment-related, 52 (4%) were from other causes, and the cause of death for the remaining seven (<1%) is currently unknown. 70 (6%) deaths occurred within 60 days of randomisation, with no difference between the two groups.

Median strategy-failure-free survival in the ITT population was 8·4 months (IQR 5·3–11·8) in arm A and 7·4 months (5·1–12·1) in arm C, and in the per-protocol population was 9·2 months (7·0–12·9) in arm A and 8·7 months (5·9–13·4) in arm C. From the Kaplan-Meier plots (figure 3), intermittent therapy seemed to result in a greater loss of patients (in terms of strategy) early on, especially around 6–9 months, but at 2 years there was little difference in number of patients remaining on strategy: 2-year survival in the ITT analysis was 7·4% (n=28) in the continuous treatment arm and 7·6% (n=35) in the intermittent treatment arm, and in the per-protocol analysis was 7·7% on continuous (n=18) and 7·6% on intermittent (n=24) treatment.

Among patients in the per-protocol population allocated intermittent treatment who commenced a chemotherapy-free interval (n=511), the median time to progression was 12·9 weeks (3·0 months; IQR 10·9–24·0 weeks). Among those who later restarted trial therapy (n=325), the median overall length of the chemotherapy-free interval before progression was 16·0 weeks (3·7 months; IQR 13·7–26·0 weeks). The median number of chemotherapy-free intervals was two (range one to six).

Protocol treatment in both treatment groups was identical up to 12 weeks (the time of the first scheduled radiological disease assessment). 192 (12%) patients in the ITT population had progressive disease at 12 weeks (table 2). Best overall response (by RECIST criteria) did not differ between treatment groups, suggesting that most patients achieve best response within the first 12 weeks of therapy. Among the 268 patients allocated intermittent treatment who restarted a second 12-week course of chemotherapy after a chemotherapy-free interval and had a second response assessment 12 weeks later, 87 (32%) had a partial response, 101 (38%) had stable disease, and 80 (30%) had progressive disease.

**Table 2:** Response at 12 weeks and best response in arms A and C and response after rechallenge (arm C only)

	**Arm A**		**Arm C**		
	12 weeks (N=815)	Best overall (N=815)	12 weeks (N=815)	Best overall (N=815)	Best after rechallenge (N=325)
Complete response	18 (2%)	40 (5%)	13 (2%)	22 (3%)	0
Partial response	339 (42%)	377 (46%)	365 (45%)	399 (49%)	88 (27%)
Stable disease*	198 (24%)	184 (23%)	196 (24%)	200 (25%)	103 (32%)
Progressive disease	96 (12%)	113 (14%)	96 (12%)	112 (14%)	77 (24%)
Missing	12 (1%)	0	9 (1%)	0	11 (3%)
Not assessed	152 (19%)	101 (12%)	136 (17%)	82 (10%)	46 (14%)

Data are n (%).

* In the COIN dataset, the first disease response assessment was at around 12 weeks; therefore, patients with stable disease must have (at least) maintained this stability for that length of time.

Haematological toxic effects and hand–foot syndrome were more common on continuous treatment than on intermittent treatment, whereas nausea and vomiting occurred more often in patients receiving intermittent treatment. Grade 3 or worse peripheral neuropathy and hand–foot syndrome were more frequent during continuous treatment (webappendix p 4).

There were fewer eligible patients in arm C (79%, n=647) than in arm A (89%, n=724; p<0·0001), since more patients in arm C either remained on trial therapy (6% *vs* 1% in arm A, n=46 *vs* nine, p<0·0001) or died (14% *vs* 9% in arm A, n=115 *vs* 74, p=0·0015). Among patients judged eligible to receive second-line therapy, this treatment was given to significantly fewer patients on intermittent therapy (52%, n=336) than on continuous therapy (62%, n=448; p=0·00020).

Quality of life in the per-protocol population at baseline, 12 weeks, and 24 weeks was assessable in 279 (60%) patients on continuous treatment and 282 (55%) patients on intermittent treatment. Less than two-thirds of patients complied with the quality-of-life component of COIN. No clear evidence of differences in baseline characteristics was identified between those patients who completed quality-of-life questionnaires and all patients randomly allocated to treatment groups, but the risk of such differences cannot be eliminated. After adjustment for previous measurements, there were significant benefits from intermittent therapy at 24 weeks for fatigue, dry or sore mouth, problems eating and drinking, difficulty handling small objects, interference with daily activities, nausea or vomiting, appetite loss, constipation, and diarrhoea (table 3; p<0·05 for all). Intermittent therapy also had benefits in terms of role functioning (p=0·015) and social functioning (p=0·016; table 3). By contrast, the only significant factor suggesting detriment from intermittent therapy was the symptom scale of pain (p=0·00029; table 3). At 12 weeks, there was a non-significant trend towards an adverse effect of intermittent therapy on emotional functioning (p=0·086), but this result was not evident at 24 weeks (p=0·90; table 3).

**Table 3:** Quality of life at 12 and 24 weeks

	**12 weeks**		**24 weeks**	
	OR (95% CI)	p value	OR (95% CI)	p value
**Functional scales**				
Impaired physical functioning	1·13 (0·97–1·30)	0·11	0·99 (0·83–1·18)	0·89
Impaired role functioning	1·11 (0·97–1·27)	0·13	0·82 (0·70–0·96)	0·015
Impaired emotional functioning	1·14 (0·98–1·31)	0·086	1·01 (0·85–1·20)	0·90
Impaired cognitive functioning	1·07 (0·92–1·23)	0·37	0·90 (0·76–1·07)	0·24
Impaired social functioning	1·05 (0·91–1·20)	0·52	0·82 (0·70–0·96)	0·016
**Symptom scales**				
Fatigue	1·02 (0·89–1·17)	0·75	0·73 (0·62–0·87)	0·00025
Nausea or vomiting	1·03 (0·90–1·19)	0·65	0·82 (0·68–0·98)	0·033
Pain	1·03 (0·89–1·20)	0·66	1·38 (1·16–1·64)	0·00029
Dyspnoea	1·06 (0·92–1·23)	0·40	1·00 (0·84–1·20)	0·99
Insomnia	1·10 (0·95–1·26)	0·19	0·94 (0·79–1·11)	0·44
Appetite loss	1·08 (0·94–1·25)	0·28	0·80 (0·67–0·95)	0·012
Constipation	1·09 (0·93–1·28)	0·28	0·82 (0·68–0·99)	0·037
Diarrhoea	0·98 (0·85–1·13)	0·79	0·79 (0·66–0·94)	0·008
Dry or sore mouth	1·04 (0·90–1·19)	0·62	0·66 (0·55–0·79)	<0·0001
Problems eating or drinking	1·23 (1·00–1·50)	0·045	0·63 (0·47–0·84)	0·0021
Problems handling small objects	1·12 (0·96–1·32)	0·16	0·54 (0·45–0·65)	<0·0001
Treatment interferes with daily activities	1·01 (0·89–1·16)	0·83	0·62 (0·52–0·73)	<0·0001
Treatment felt to have been worthwhile	0·94 (0·80–1·11)	0·46	1·20 (0·96–1·50)	0·12
**Global scales**				
Global quality of life	1·08 (0·93–1·25)	0·30	0·98 (0·83–1·15)	0·81

Odds ratios (ORs) are for arm C compared with arm A, and are from ordinal regression models adjusting for previous timepoints (baseline, 12 weeks). ORs greater than 1 indicate worse quality of life, and ORs less than 1 indicate better quality of life.

**Figure 4:**
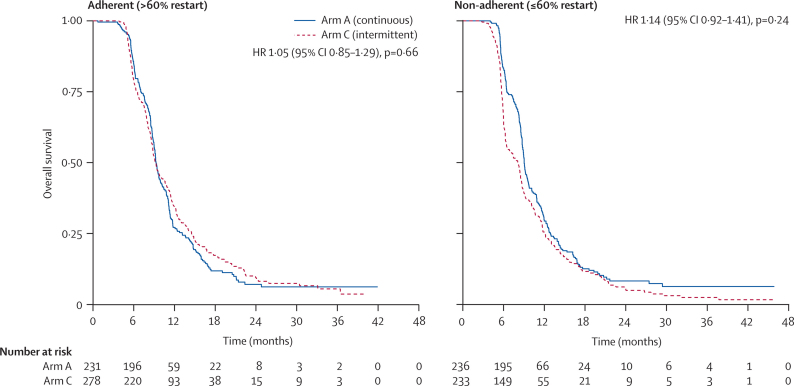
Effects of adherence to protocol on overall survival within the per-protocol population Interaction with treatment arm: hazard ratio (HR) 1·08 (95% CI 0·80–1·46); p=0·60.

We undertook a post-hoc exploratory analysis of the effects of protocol adherence on overall survival. Clinicians were classified according to the proportion of their patients assigned to intermittent therapy in the per-protocol population who restarted treatment after a chemotherapy-free interval. A cutoff of 60% was chosen because this proportion formed a pragmatic and appropriate benchmark midway between the 40% and 80% of restarts reported in OPTIMOX-1 and OPTIMOX-2, respectively. For so-called adherent clinicians, there was no survival difference between arms A and C. But for non-adherent clinicians, there was an early survival advantage for arm A. However, this difference lessens over time and the overall HR is non-significant (figure 4).

**Figure 5:**
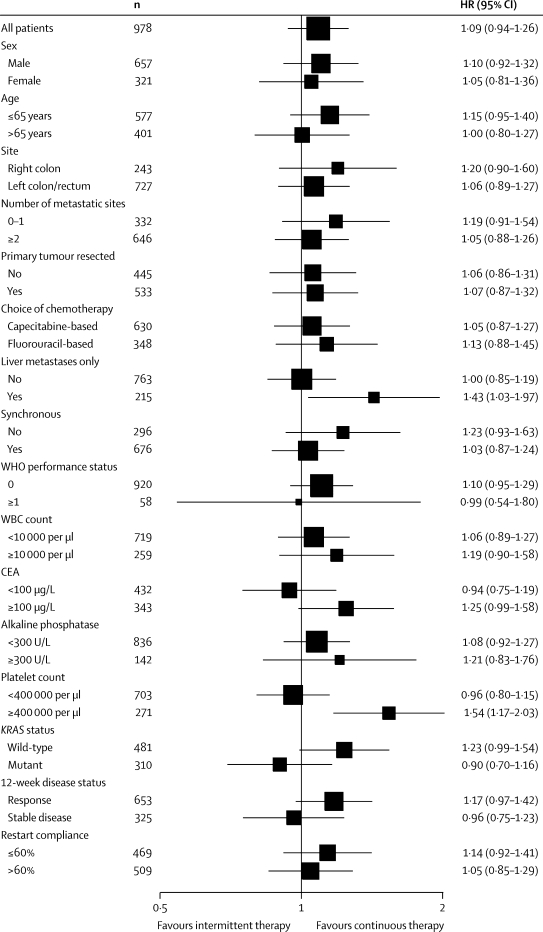
Subgroup analyses of overall survival within the per-protocol population HR=hazard ratio. WBC=white blood cell. CEA=carcinoembryonic antigen.

**Figure 6:**
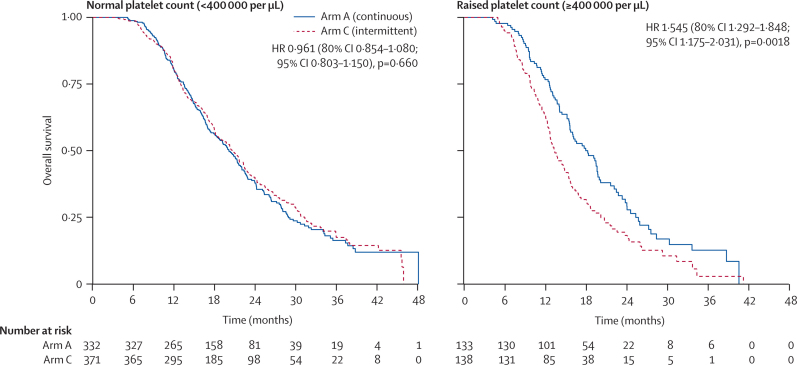
Kaplan-Meier curves of overall survival within the per-protocol population, by baseline platelet subgroup Interaction with treatment arm: hazard ratio (HR) 1·646 (95% CI 1·188–2·279); p=0·0027.

15 prespecified factors were entered into a prognostic model to predict overall survival in the per-protocol population, together with the post-hoc factor of restart adherence rate. The final model contained the following previously identified prognostic variables: previous adjuvant chemotherapy or surgery; alkaline phosphatase; body surface-area; platelet count; time from diagnosis to randomisation; resection of primary tumour; mutation present in any of *KRAS*, *NRAS,* or *BRAF*; and number of metastatic sites. In particular, each of the four variables identified by Köhne and colleagues^13^ were present with the exception of white blood cell count (in our data, platelet count was a stronger prognostic indicator), and the score defined by Köhne was strongly prognostic (p<0·0001 for trend). The 16 factors entered into the prognostic model were also explored for predictive value for overall survival in the per-protocol population (figure 5). The strongest predictive factor, and the only one with an interaction significant at the 5% level, was platelet count. A raised platelet count at baseline (≥400 000 per μL, recorded for 271 [28%] of 978 patients) predicts a significant survival detriment from intermittent chemotherapy (p=0·0027 for interaction; figure 6). Raised baseline platelet count had an even greater predictive effect for a detriment from intermittent therapy on 24-week quality of life, particularly for functional scales (p<0·05 for all), for which a consistent trend was noted towards benefit from intermittent therapy for patients with normal platelet count and an adverse effect for patients with raised platelet count. Additionally, the symptom scales of fatigue, pain, dyspnoea, appetite loss, constipation, and global quality of life followed this trend (p<0·05 for all). Furthermore, patients on intermittent therapy were significantly more likely than were those on continuous therapy to report at 24 weeks that their treatment had not been worthwhile if their baseline platelet count was raised (p=0·048 for interaction; webappendix p 7). All these results were independent of known prognostic factors. Baseline platelet count and progression between 12 and 24 weeks were not associated in this dataset (p=0·41). Other factors with interactions significant at the 10% level were presence of metastases in the liver only (p=0·066) and tumoral *KRAS* status (p=0·070).

## Discussion

The goal of palliative chemotherapy is to increase overall survival with improvement or minimum deficit in quality of life. This study provides a large dataset relevant to the effect of chemotherapy-free intervals on both overall survival and quality of life in advanced colorectal cancer. The trial did not meet its primary outcome objective, which was to show non-inferiority of intermittent chemotherapy versus continuous chemotherapy as first-line therapy in advanced colorectal cancer. This policy therefore cannot be routinely recommended for all patients. However, there seems to be a large subpopulation of patients for whom intermittent therapy provides similar survival benefit and can be recommended.

The COIN trial has shown that intermittent chemotherapy is associated with improved quality of life, shortened time on chemotherapy, reduced number of visits to hospital, and a minimum difference in overall survival. Non-inferiority trials are often misconstrued as equivalence trials, which they are not. The outcomes of such trials give an indication of similarity, which should be interpreted with all relevant data in hand and not as a single endpoint. In COIN, we developed a statistical plan that set the outer bound of the HR for non-inferiority for overall survival at the rigorous level of 1·162 (for comparison, the outer bound in the comparison of fluorouracil versus capecitabine in the X-ACT trial was 1·25).^14^ The trial did not meet this strict criterion (upper limit of HR in the ITT group was 1·165). This result does not suggest that continuous chemotherapy is superior to an intermittent strategy, but does translate into an observed difference in median overall survival of around 6 weeks (1·4 months), favouring continuous therapy. Our confidence intervals reliably exclude a detriment greater than 2·2 months (ITT analysis) or 3·2 months (per-protocol analysis) in median overall survival with intermittent therapy.

The striking outcome from the study is the significant correlation of a raised baseline platelet count as a predictive biomarker for use of a continuous or intermittent chemotherapy strategy. We analysed a full range of prognostic factors including those in the Köhne prognostic index^13^ to assess correlation with outcome. A quarter of patients had a raised platelet count at baseline and these patients had substantially inferior survival (a 5-month reduction in survival; test for interaction p=0·0027) when treated with intermittent chemotherapy and also impaired quality of life on almost all scales apart from those directly related to toxic effects of chemotherapy. By contrast, the three-quarters of patients with normal platelet counts had improved quality of life on almost all measures, with no detriment in overall survival. Raised platelet count has previously been identified as a poor prognostic marker in advanced colorectal cancer.^13^ The mechanism could relate to paracrine feedback driven by cytokines including interleukin 6, directly causing thrombocytosis.^15^ It could, alternatively, relate to an aspect of immunomodulation driven by T-regulatory cells and FOXP3 epigenetic regulation.^16^ If confirmed in other datasets, the easily measurable marker of a raised platelet count at initiation of chemotherapy would be a helpful and cost-effective predictive biomarker for identification of patients in whom continuous therapy might be preferable in order to maximise symptom control and survival.

The shorter overall survival in this UK trial compared with some other trial settings is probably related to a combination of several factors, such as a more advanced distribution of disease at presentation, fewer available effective treatments, or different attitudes of patients and clinicians to additional therapy. A recent report^17^ shows from registry data that survival for colorectal cancer is lower in the UK than for other comparable countries (for 1-year survival UK registries reported 75% compared with 80–85% for the other registries in the comparable years 2005–07; 54% *vs* 58–66% at 5 years). The patterns are consistent with later stage at diagnosis or differences in treatment, particularly in Denmark and the UK. Since COIN recruited widely from 111 centres across the UK and Ireland and had broad inclusion criteria, it is likely to be representative of outcomes for advanced colorectal cancer in the UK.

Failure to restart oxaliplatin-based chemotherapy after an interval has previously been identified as detrimental to survival.^18^ In the COIN per-protocol population (ie, patients clinically eligible to recommence chemotherapy), 325 (64%) of 511 potentially eligible patients restarted a second 12-week course of chemotherapy. This proportion compares with a reintroduction rate of 40% in OPTIMOX-1^7^ and 80% in OPTIMOX-2, which recruited patients from only 12 centres and in which high rates of reintroduction were protocol mandated.^8^ The figure of 64% from the COIN trial could reasonably represent a good reflection of real practice in an incurable disease, in which chemotherapy must strike a balance between quality and length of life. The proportion of COIN patients restarting oxaliplatin and the low numbers receiving second-line chemotherapy regimens compared with some other countries reflects a less aggressive approach that is characteristic of UK oncology and could also have contributed to the lower overall survival.

Additionally, the predominantly UK study setting is important in that bevacizumab was not reimbursed for first-line treatment or subsequent use in routine NHS practice during the study period, and although its availability would probably have resulted in longer overall survival, the effect of addition of this targeted agent on a chemotherapy-free interval strategy is unclear. Three randomised trials are examining this issue,^19^, ^20^, ^21^ but preliminary data are only available for one. The TTD MACRO trial^19^ assessing continuous oxaliplatin, capecitabine, and bevacizumab versus intermittent chemotherapy with maintenance bevacizumab demonstrated non-inferiority by their criteria in terms of median time to first progression of 11·0 versus 10·3 months (HR 1·07, 0·84–1·36), but overall survival outcomes are awaited. The two other trials continue to recruit. In the AIO trial,^20^ patients are randomly allocated to one of three arms (bevacizumab plus fluoropyrimidine, bevacizumab alone, or no treatment after 24 weeks of induction oxaliplatin fluoropyrimidine and bevacizumab) and complete accrual is due in 2013. In the CAIRO 3 study,^21^ patients are randomly assigned to bevacizumab plus capecitabine or no treatment after 18 weeks of induction oxaliplatin, capecitabine, and bevacizumab.

EGFR-targeted monoclonal antibodies (cetuximab and panitumumab) have also been recently licensed in the first-line advanced colorectal cancer setting and cetuximab has been explored in an intermittent strategy in the Nordic VII^22^ and COIN-B trials.^23^ NORDIC VII demonstrated median overall survival of 20·4, 19·7, and 20·3 months in the continuous chemotherapy alone, continuous chemotherapy plus cetuximab, and intermittent chemotherapy plus continuous cetuximab arms, respectively, which were not statistically different.^22^ Preliminary results from the COIN-B trial are expected during 2011. Consequently, our findings should be interpreted cautiously for health-care environments in which bevacizumab, cetuximab, and panitumumab are widely available. However, our message that omission of chemotherapy for long periods in selected patients without overall survival deficit could be even more relevant in such contexts. Potential benefits from the continuation of such targeted agents with or without the addition of a fluoropyrimidine, when oxaliplatin is temporarily discontinued, have yet to be shown in terms of progression-free or strategy-failure-free survival or more importantly in terms of overall survival in any completed randomised controlled trials; such data are eagerly awaited.

Standard UK practice is to image patients at 12-week intervals. International trials, especially commercially sponsored studies evaluating the effects of novel agents, now often use assessments every 6–8 weeks. Although COIN only required imaging every 12 weeks, clinical assessment was done every 6 weeks, during which clinical or biochemical progression would have been identified. During treatment breaks, patients in arm C attended hospital as outpatients or day cases on an average of two occasions every 12–24 weeks, whereas those in arm A would have been seen on at least eight occasions and possibly 12 depending on the chemotherapy regimen. This more frequent clinical review could have led to earlier identification of disease progression.

The secondary and subgroup analyses of the trial are intended to offer some guidance to practising clinicians, patients, and patient advocates. Against the trend to marginally reduced survival with intermittent chemotherapy is set reduced time on chemotherapy (on average 2·3 months less) and less frequent hospital attendances. There is also evidence of reduced cumulative toxicities of neuropathy and hand–foot syndrome. The overall effect on quality of life with time off treatment in arm C, as assessed at the specific timepoints of 12 and 24 weeks, suggest an improvement in fatigue, nausea and vomiting, anorexia or loss of appetite, constipation, diarrhoea, dry or sore mouth, and difficulty handling objects. However, pain seems to be slightly but consistently more frequent in those receiving the intermittent strategy, which could be accounted for by the increased likelihood of tumour progression or activity during a chemotherapy-free interval. By contrast, emotional functioning appeared worse in the intermittent group at the end of the first 12-week treatment period when all patients had received equivalent therapy. This result might reflect anxiety induced by the idea of treatment being stopped temporarily. Yet this finding disappears at 24 weeks, when those on continuous therapy may be uncertain about continuing effectiveness or tolerability of ongoing treatment.

In advanced colorectal cancer, for patients treated with palliative intent, as in most other malignancies, time off chemotherapy remains a treatment option. This study has shown and quantified the benefits of a chemotherapy-free interval in terms of reduced time on chemotherapy, reduced cumulative toxic effects, and improved quality of life after an initial 12-week period of induction chemotherapy. In the whole population, there was a small reduction in overall survival with intermittent compared with continuous chemotherapy, exceeding our predefined non-inferiority bound. However, the identification of raised platelet count as a potential biomarker separating patient subsets who do better from those who do not is very important. Thrombocytosis could identify a patient subgroup in whom cytokine activation is driving a more aggressive disease course, but which is sensitive to therapeutic intervention. By contrast, the three-quarters of patients in COIN with normal platelet counts at randomisation suffered no loss in overall survival and could reap potentially important benefits of chemotherapy-free intervals.

Panel **Research in context**

**Systematic review**

Conventional palliative chemotherapy for treatment of advanced colorectal cancer is given continuously until progressive disease or cumulative toxic effects occur, or the patient chooses to discontinue. In a previous Medical Research Council study, CR06B,^5^ a shortened course of chemotherapy with reuse of the same treatment on progression showed an improvement in quality of life with no loss of survival. Similar findings have been reported in other cancer types, generally showing that short-course treatment is as effective as long-course. One consistent finding is that patients receiving short-course therapy can have a reduced time to disease progression, but several investigators have reported that at the time of progression patients can be successfully rechallenged with the same regimen. Searches of Medline and Cancerlit for publications and PDQ (Physicians Data Query) and the UKCCCR trial register for any additional open or closed trials in advanced colorectal cancer found no trials comparing these approaches when this trial was started.

**Interpretation**

Survival in COIN is shorter than for many international trials in advanced colorectal cancer, which probably reflects a more advanced stage of disease at presentation in the UK. Additionally, the lack of routine availability of biological agents in the UK National Health Service might contribute to shortened survival and therefore restrict direct application of the COIN data in the most resource-rich health-care settings. The finding that intermittent chemotherapy is inferior in patients with raised platelet counts is novel and needs confirmation, but supports the conventional practice of continuous chemotherapy for this cohort. Conversely, for most patients with normal platelet counts at baseline, this study suggests that chemotherapy-free intervals are associated with improved quality of life and no loss of overall survival and is therefore an option that clinicians should discuss with such patients.

## Web Extra Material

Supplementary webappendix
