# Supplementary material for: Intermittent versus continuous oxaliplatin and fluoropyrimidine combination chemotherapy for first-line treatment of advanced colorectal cancer: results of the randomised phase 3 MRC COIN trial
Source: Lancet Oncol. 2011 Jun 4;12(7):642–53. doi: 10.1016/S1470-2045(11)70102-4 (PMC3159416; doi:10.1016/S1470-2045(11)70102-4)
Supplement: Supplementary webappendix [file mmc1.pdf]

THE LANCET Oncology

Supplementary webappendix

This webappendix formed part of the original submission and has been peer reviewed. We post it as supplied by the authors.

Supplement to: Adams RA, Meade AM, Seymour MT, et al, on behalf of the MRC COIN Trial Investigators. Intermittent versus continuous oxaliplatin and fluoropyrimidine combination chemotherapy for first-line treatment of advanced colorectal cancer: results of the randomised phase 3 MRC COIN trial. *Lancet Oncol* 2011; published online June 4. DOI:10.1016/S1470-2045(11)70102-4.

Web Appendix

Title: "Intermittent versus continuous oxaliplatin and fluoropyrimidine combination chemotherapy in the first-line treatment of patients with advanced colorectal cancer: results of the randomised phase 3 MRC COIN trial."

List of Clinical Investigators (Institution – [number of patients contributed]):
C Lowdell, RH Phillips, R Ahmad, P Riddle, A Creak (Charing Cross Hospital, London, UK [81]); J Cassidy, A McDonald, A Waterston, N Mohammed, J White, H Yosef, A Hennessy (Beatson Oncology Centre, Glasgow, UK [81]); EM Bessell, V Potter (Nottingham City Hospital, Nottingham, UK [72]); M Seymour, A Anthoney, A Melcher, R Cooper, D Sebag-Montefiore (St. James' University Hospital, Leeds, UK [69]); P Ross, M Leslie, N Maisey, A Gaya, G Mikhaeel (Guy's and St. Thomas's Hospitals, London, UK [67]); J Summers, M Hill, R James (Maidstone Hospital, Maidstone, UK [64]); TS Maugham, A Brewster, T Crosby, S Mukherjee, N Iqbal (Velindre Hospital, Cardiff, UK [63]); J Wadsley, D Furniss, S Pledge, J Hornbuckle, S Clenton (Weston Park Hospital, Sheffield, UK [63]); R Glynne-Jones, M Harrison, S Mawdsley, N Anyamene, R Hughes (Mount Vernon Hospital, UK [57]); D Propper, C Cottrill (St. Bartholomew's Hospital, London, UK [57]); D Smith, S Myint, N Ali, M Iqbal, P Clark, B Haylock (Clatterbridge Centre for Oncology, UK [50]); T Iveson, A Bateman, C Baughan (Southampton Hospital, UK [46]); S Falk, K Hopkins (Bristol Oncology Centre, Bristol, UK [45]; J Bridgewater, S Karp (North Middlesex Hospital, London, UK [44]); C Topham, G Middleton, S Essapen, S Cummins (Royal Surrey County Hospital, UK [44]); B Sizer (Essex County Hospital, UK [42]); S Gollins (Glan Clwyd Hospital, UK [42]); A Maraveyas (Castle Hill Hospital, Hull, UK [41]); S Tahir (Broomfield Hospital, Essex, UK [36]); J Bridgewater (Princess Alexandra Hospital, UK [36]; C Blesing (Great Western Hospital, Swindon, UK [34]); H Wasan (Hammersmith Hospital, London, UK [34]); M Tighe, S Falk (Musgrove Park Hospital, Taunton [34]); R Ellis (Royal Cornwall Hospital, UK [34]); J Dent, JK Joffe (Huddersfield Royal Infirmary, UK [33]); S Shepherd, K Benstead, D Farrugia (Cheltenham General Hospital, UK [32]); P Mack, A Hamid, M Butt, R Roy (Diana, Princess of Wales Hospital, Grimsby, UK [31]); T Hickish (Royal Bournemouth Hospital, UK [31]); H Yosef (Hairmyres Hospital, UK [29]); D Tsang, P Leonard, J Prejbisz (Southend Hospital, UK [28]); J Ledermann, J Bridgewater (University College London Hospital, London, UK [27]); T Hickish, R Osborne (Poole Hospital, Dorset, UK [26]); A O'Callaghan, SR Muthuramalingam (Portsmouth Oncology Centre, Queen Alexandra Hospital, UK [25]); F Adab (University Hospital of North Staffordshire NHS Trust, UK [25]); NJ Wadd, J Van der Voet, D Wilson (James Cook University Hospital, UK [24]); (Singleton Hospital, UK [24]); I Pedley, A Azzabi (Sunderland Royal Hospital, UK [24]); E Marshall (Whiston Hospital, UK [24]); R James (Kent and Canterbury Hospital, UK [23]); F Daniel (Derriford Hospital, Plymouth, UK [22]); R Osborne (Dorset County Hospital, UK [22]); TJ Iveson (Salisbury District Hospital, UK [22]); D Jodrell, C McLean, S Clive, L Dawson, HA Philips (Western General Hospital, Edinburgh, UK [22]); S Falk (Yeovil District Hospital, UK [22]); R James (Queen Elizabeth, The Queen Mother Hospital, UK [21]); P Chakraborti, R Kulkarni (Royal Derby Hospital, UK [21]); LM Samuel, G MacDonald (Aberdeen Royal Infirmary, UK [20]); C Bradley, C Twelves (Bradford Royal Infirmary, UK [20]); S Giridharan, F Adab (Staffordshire General Hospital, UK [20]); S Myint (Southport & Formby District General Hospital, UK [18]); S Gollins (Wrexham Maelor Hospital, UK [18]); N Stuart, C Bale (Ysbyty Gwynedd Hospital, UK [18]); (William Harvey Hospital, UK [17]); J Valle, M Saunders, G Wilson (Christie Hospital, Manchester, UK [16]); A Weaver, A Jones (Churchill Hospital, Oxford, UK [16]); K McAdam, C Jephcott (Peterborough District Hospital, UK [16]); S Cleator (St. Mary's Hospital, London, UK [16]); (Worthing Hospital, UK [16]); J Glaholm (Good Hope Hospital, UK [15]); R Hughes, PM Mulholland (Lister Hospital, UK [15]); N Steven (Queen Elizabeth, University Hospital, Birmingham, UK [15]); A Mayer, T Meyer (Royal Free Hospital [15]); N Lo, G Cogill, L Toy (Torbay Hospital, UK [15]); M Wilkins (West Wales General Hospital, UK [15]); C Wilson, C Palmer (Addenbrooke's Hospital, UK [14]); I Geh (Birmingham Heartlands Hospital, UK [14]); R Thomas (Bedford Hospital NHS Trust [13]); J Nicoll (Cumberland Infirmary, UK [13]); P Chakraborti (Queens Hospital, Burton Upon Trent [13]); A Azzabi (South Tyneside Hospital, UK [13]); R Wilson, M Eatock (Belfast City Hospital, UK [12]); A Hartley (Manor Hospital, Walsall, UK [12]); J Gildersleve, A Freebairn (Berkshire Cancer Centre, Berkshire, UK [12]); GP Deutsch, A Webb, M Wilkins (Royal Sussex County Hospital, Brighton, UK [12]); F McKinna (Eastbourne District General Hospital [11]); D Cunningham, I Chau (Royal Marsden, Sutton, UK [11]); S Susnerwala, M Wise, A Birtle (Royal Preston Hospital, UK [11]); A Hamid (Scunthorpe General Hospital, UK [11]); FJ Lofts (St. George's Hospital, London, UK [11]); J Kennedy (St. James' Hospital, Dublin, Republic of Ireland [11]); D Whillis (Raigmore Hospital, UK [10]); S Susnerwala (Blackpool Victoria Hospital, UK [9]); LT Tan, C Palmer (Hinchingbrooke Hospital, UK [9]); N Maisey (Queen Elizabeth Hospital, UK [9]); M Keane (University College Hospital, Galway, Republic of Ireland [9]); C Macmillan, K Patel (Northampton General Hospital, UK [8]); D Farrugia (Worcestershire Royal Hospital, UK [8]); G Bertelli, V Vigneswaran (Withybush General Hospital, UK [7]); D Smith (Aintree, University Hospital, UK [6]); R McDermott (AMNCH, Tallaght Hospital, Dublin, Republic of Ireland [6]); S O'Reilly (Mercy Hospital, Cork, Republic of Ireland [6]); V Hall (Royal Hampshire County Hospital, Winchester, UK [6]); C

Hamilton, A Dhadda (Scarborough Hospital, UK [6]); C Baughan (St. Mary's Hospital, Isle Of Wight, UK [6]); H Ford, M Moody (West Suffolk Hospital, UK [6]); M Tomlinson (Weston General Hospital, Weston Super Mare, UK [6]); L Grogan, O Breathnach (Beaumont Hospital, Dublin, Republic of Ireland [4]); R Soomal (Ipswich Hospital NHS Trust, UK [4]); J McCaffrey (Mater Misericordiae Private Hospital, Dublin, Republic of Ireland [4]); J McCaffrey (Mater Misericordiae (Public) Hospital, Dublin, Republic of Ireland [4]); C Rees (North Hampshire Hospital, Basingstoke, UK [4]); PJ Atherton (North Tyneside General Hospital, UK [4]); S Beesley (Conquest Hospital, UK [3]); W Dobrowsky (Wansbeck General Hospital, UK [3]); G Leonard (Waterford Regional Hospital, Waterford, Republic of Ireland [3]); R Gupta (Midwestern Regional Hospital, Limerick, Republic of Ireland [2]); J Stewart (Milton Keynes General Hospital, UK [2]); D Cunningham (Royal Marsden Hospital, Fulham Road, London, UK [2]).

In addition to the above-named individuals, we acknowledge the contributions of a large number of clinicians, research nurses, data managers and other clinical and support staff at the participating centres.

Definition of "Strategy Failure-Free Survival" SFFS

Arm A

- Identical to Progression-Free Survival (PFS); that is, the earliest of:
    - Date of disease assessment if status is progressive disease (PD)
    - Date of COIN treatment failure if for progression or death
    - Date of death

Arm C

- Date of disease assessment if status is PD and patient is on treatment
- Date of disease assessment if status is PD and patient is *off* treatment, if one of the following also applies:
    - within 8 weeks of coming off treatment (that is, so soon after coming off treatment that PD probably occurred while on treatment)
    - over 8 weeks off treatment but treatment does not recommence within one month (that is, failure of protocol with regard to re-starting treatment)
- Date of COIN treatment failure if for progression or death, whether on or off treatment

Censoring:

- Date last seen
    - That is, latest recorded date of treatment cycle, toxicity/disease assessment, follow-up, date of COIN treatment failure, death, or randomisation

Supplementary Tables

Table 1: Rates of G3+ toxicity symptoms (CTC v3.0) experienced after 12-week assessment by the PPA population

	Arm A		Arm C		
	N	(%)	N	(%)	p-value
**Haematological toxicities**					
Platelets	13	(3%)	7	(1%)	P=0.12
Haemoglobin	6	(1%)	15	(3%)	P=0.075
WBC	16	(3%)	11	(2%)	P=0.23
Neutrophils	56	(12%)	40	(8%)	P=0.029
**Non-haematological toxicities**					
Nausea	9	(2%)	34	(7%)	P=0.0032
Vomiting	8	(2%)	21	(4%)	P=0.027
Diarrhoea	38	(8%)	50	(10%)	P=0.37
HFS	21	(5%)	15	(3%)	P=0.20
Nail changes	0	(0%)	0	(0%)	--
Skin rash	0	(0%)	2	(<1%)	P=0.50
Peripheral neuropathy	126	(27%)	25	(5%)	P<0.0001
Hypomagnesaemia	0	(0%)	4	(1%)	P=0.13
Anorexia	13	(3%)	28	(5%)	P=0.036
Alopecia	2	(<1%)	1	(<1%)	P=0.61
Pain	42	(9%)	81	(16%)	P=0.0012
Stomatitis	6	(1%)	5	(1%)	P=0.77
Lethargy	68	(15%)	95	(19%)	P=0.091
Vein pain	1	(<1%)	3	(1%)	P=0.63
**Total**	**467**	**(100%)**	**511**	**(100%)**	

Table 2: Baseline characteristics by availability of QoL data, PPA population

	QoL data available at BL, 12wks and 24wks? No N	(%)	Yes N	(%)	Total N	(%)	p-value
**Treatment arm**							
Arm A	188	(45%)	279	(50%)	467	(48%)	
Arm C	229	(55%)	282	(50%)	511	(52%)	P=0.15
**Chemo regimen**							
XELOX	274	(66%)	356	(63%)	630	(64%)	
OxMdG	143	(34%)	205	(37%)	348	(36%)	P=0.47
**Sex**							
Male	280	(67%)	377	(67%)	657	(67%)	
Female	137	(33%)	184	(33%)	321	(33%)	P=0.99
**Age at randomisation**							
<=65y	239	(57%)	338	(60%)	577	(59%)	
>65y	178	(43%)	223	(40%)	401	(41%)	P=0.36
**WHO PS**							
0	194	(47%)	290	(52%)	484	(49%)	
1	194	(47%)	242	(43%)	436	(45%)	
2	29	(7%)	29	(5%)	58	(6%)	P=0.20
**Location of primary tumour**							
Right colon	110	(26%)	133	(24%)	243	(25%)	
Left colon/rectum	305	(73%)	422	(76%)	727	(75%)	P=0.37
**Status of primary tumour**							
Resected	219	(53%)	314	(56%)	533	(56%)	
Unresected	177	(42%)	226	(40%)	403	(41%)	
Local recurrence	21	(5%)	21	(4%)	42	(4%)	P=0.42
**Liver-only metastases**							
No	323	(77%)	440	(78%)	763	(78%)	
Yes	94	(23%)	121	(22%)	215	(22%)	P=0.72
**No. of metastatic sites**							
0/1	150	(36%)	182	(32%)	332	(34%)	
2+	267	(64%)	379	(68%)	646	(66%)	P=0.25
**Timing of metastases**							
Metachronous	122	(29%)	174	(31%)	296	(30%)	
Synchronous	291	(70%)	385	(69%)	676	(70%)	P=0.59
**Total**	**417**	**(100%)**	**561**	**(100%)**	**978**	**(100%)**	

	QoL data available at BL, 12wks and 24wks?						
	No		Yes		Total		
	N	(%)	N	(%)	N	(%)	p-value
**Prior radiotherapy**							
No	407	(98%)	548	(98%)	955	(98%)	
Yes	10	(2%)	13	(2%)	23	(2%)	P=0.93
**Prior surgery**							
No	340	(82%)	458	(82%)	798	(82%)	
Yes	77	(18%)	103	(18%)	180	(18%)	P=0.97
**Adjuvant chemo/radio**							
No	307	(74%)	411	(73%)	718	(73%)	
Yes	110	(26%)	150	(27%)	260	(27%)	P=0.90
**Size of centre**							
Small	142	(34%)	193	(34%)	335	(34%)	
Medium	138	(33%)	222	(40%)	360	(37%)	
Large	137	(33%)	146	(26%)	283	(29%)	P=0.037
**WBC**							
<10,000/l	307	(74%)	412	(73%)	719	(74%)	
>=10,000/l	110	(26%)	149	(27%)	259	(26%)	P=0.95
**Alk phos**							
<300 U/l	350	(84%)	486	(87%)	836	(86%)	
>=300 U/l	67	(16%)	75	(13%)	142	(14%)	P=0.24
**Platelet count**							
<400,000/l	294	(71%)	409	(73%)	702	(72%)	
>=400,000/l	120	(29%)	151	(27%)	270	(28%)	P=0.49
**Total**	**417**	**(100%)**	**561**	**(100%)**	**978**	**(100%)**	

Table 3: Effect of baseline platelet count on 24-week QoL, PPA population

	Normal platelet count (<400,000/l)			Raised platelet count (≥400,000/l)			Interaction
	OR *	95% CI	p-value	OR *	95% CI	p-value	p-value
**Functional scales**							
Physical functioning	0.83	(0.67, 1.02)	P=0.079	1.44	(1.04, 2.00)	P=0.029	P=0.0020
Role functioning	0.72	(0.59, 0.87)	P=0.00063	1.15	(0.85, 1.56)	P=0.37	P=0.0064
Emotional functioning	0.90	(0.73, 1.11)	P=0.33	1.32	(0.96, 1.82)	P=0.085	P=0.030
Cognitive functioning	0.79	(0.64, 0.98)	P=0.034	1.19	(0.86, 1.64)	P=0.31	P=0.045
Social functioning	0.70	(0.58, 0.85)	P=0.00031	1.17	(0.86, 1.59)	P=0.31	P=0.0046
**Symptom scales**							
Fatigue	0.60	(0.49, 0.74)	P<0.0001	1.19	(0.86, 1.65)	P=0.28	P=0.00094
Nausea/vomiting	0.73	(0.59, 0.92)	P=0.0073	1.00	(0.70, 1.43)	P=0.99	P=0.12
Pain	1.18	(0.96, 1.46)	P=0.12	2.14	(1.54, 2.98)	P<0.0001	P=0.0015
Dyspnoea	0.89	(0.72, 1.1`)	P=0.31	1.38	(0.98, 1.94)	P=0.066	P=0.045
Insomnia	0.90	(0.74, 1.11)	P=0.34	1.04	(0.77, 1.41)	P=0.81	P=0.37
Appetite loss	0.69	(0.55, 0.86)	P=0.00075	1.11	(0.81, 1.52)	P=0.53	P=0.018
Constipation	0.65	(0.52, 0.83)	P=0.00034	1.36	(0.95, 1.93)	P=0.092	P=0.0012
Diarrhoea	0.75	(0.61, 0.93)	P=0.0092	0.84	(0.60, 1.18)	P=0.32	P=0.82
Dry/sore mouth	0.59	(0.47, 0.74)	P<0.0001	0.83	(0.59, 1.17)	P=0.28	P=0.15
Problems eating/drinking	0.55	(0.37, 0.82)	P=0.0033	0.71	(0.44, 1.15)	P=0.16	P=0.37
Problems handling small objects	0.51	(0.41, 0.64)	P<0.0001	0.62	(0.44, 0.89)	P=0.010	P=0.58
Trt interferes with daily activities	0.56	(0.46, 0.69)	P<0.0001	0.82	(0.59, 1.14)	P=0.23	P=0.15
Trt has been worthwhile	1.04	(0.80, 1.36)	P=0.76	1.68	(1.05, 2.69)	P=0.031	P=0.048
**Global scales**							
Global QoL	0.86	(0.71, 1.05)	P=0.13	1.36	(0.99, 1.87)	P=0.059	P=0.012

* Odds ratios are for Arm C compared to Arm A, and are from ordinal regression models adjusting for previous timepoints (baseline, 12 weeks). ORs >1 indicate worse quality of life, and ORs <1 indicate better quality of life.
